# Automated parameterisation for multi-scale image segmentation on multiple layers

**DOI:** 10.1016/j.isprsjprs.2013.11.018

**Published:** 2014-02

**Authors:** L. Drăguţ, O. Csillik, C. Eisank, D. Tiede

**Affiliations:** a Department of Geography, West University of Timişoara, V. Pârvan Blv. 4, 300223 Timişoara, Romania; b Interfaculty Department of Geoinformatics – Z_GIS, University of Salzburg, Schillerstraße 30, 5020 Salzburg, Austria

**Keywords:** Automation, Imagery, Object, Representation, GEOBIA, MRS

## Abstract

We introduce a new automated approach to parameterising multi-scale image segmentation of multiple layers, and we implemented it as a generic tool for the eCognition® software. This approach relies on the potential of the local variance (LV) to detect scale transitions in geospatial data. The tool detects the number of layers added to a project and segments them iteratively with a multiresolution segmentation algorithm in a bottom-up approach, where the scale factor in the segmentation, namely, the scale parameter (SP), increases with a constant increment. The average LV value of the objects in all of the layers is computed and serves as a condition for stopping the iterations: when a scale level records an LV value that is equal to or lower than the previous value, the iteration ends, and the objects segmented in the previous level are retained. Three orders of magnitude of SP lags produce a corresponding number of scale levels. Tests on very high resolution imagery provided satisfactory results for generic applicability. The tool has a significant potential for enabling objectivity and automation of GEOBIA analysis.

## 1 Introduction

Geographic object-based image analysis (GEOBIA) has been gaining prominence in the fields of remote sensing and geographic information science (GIScience) over the past decade, especially for the processing of high spatial resolution imagery (Blaschke, 2010). Creating representative image objects with image segmentation algorithms is an important pre-requisite for classification/feature extraction and further integration in geographical information systems (GIS) analysis. Multiresolution Segmentation (MRS) (Baatz and Schäpe, 2000) is probably the most popular algorithm for these purposes. Implemented in the eCognition® software (Trimble Geospatial Imaging), this algorithm quickly became one of the most important segmentation algorithms within the GEOBIA domain. MRS relies on a key control, called the scale parameter (SP), to partition an image into image objects. The SP controls the internal (spectral) heterogeneity of the image objects and is therefore correlated with their average size, i.e., a larger value of the SP allows a higher internal heterogeneity, which increases the number of pixels per image-object (Baatz and Schäpe, 2000, Benz et al., 2004).

Because the average size of image objects critically impacts on the classification accuracy (Gao et al., 2011), the selection of an accurate value of the SP is a crucial decision in segmenting remote sensing imagery (Kim et al., 2011). However, the standard procedure that leads to this decision is a trial-and-error optimisation (e.g. Duro et al., 2012), which is based on a visual assessment of segmentation suitability (Whiteside et al., 2011). While allowing flexibility in incorporating expert knowledge in GEOBIA, this procedure is hardly reproducible and raises important scientific issues with respect to the robustness of the approach (Arvor et al., 2013).

Since the SP is the key control in MRS and heavily impacts on the classification accuracy, making its selection a more objective decision (at least traceable or reproducible) is a hot topic in GEOBIA (Blaschke, 2010). According to Zhang et al. (2008), methods of evaluating the image segmentation quality to identify suitable segmentation parameters can be classified into supervised and unsupervised, aside from the standard visual assessment. Unsupervised methods can lead to the self-tuning of segmentation parameters, which is, thus, automation (Zhang et al., 2008). The concept of local variance (LV) graphs (Woodcock and Strahler, 1987) was introduced to GEOBIA by Kim et al. (2008) to determine the optimal SP for alliance-level forest classification of multispectral IKONOS images. Drăguţ et al. (2010) automated this approach and extended it into multi-scale analysis based on single layers and created a generic tool to detect the scales where patterns occur in the data, which is called the Estimation of Scale Parameters (ESP tool). Espindola et al. (2006) proposed an objective function that obeys the principles of regionalisation, namely, minimising the internal variance while maximising the external difference. Martha et al. (2011) further developed this approach into multi-scale analysis. Johnson and Xie (2011) employed the same heterogeneity measures (weighted variance and Moran’s I, respectively) to identify and further refine over- and under-segmented regions within a given scale level.

All of the above-mentioned existing methods require user interpretation, which hinders automation of the segmentation and of the rule-sets in a GEOBIA framework. Udupa et al. (2006) argued that segmentation methods cannot be automatic, which might be true when segmentation does necessarily include object recognition. In GEOBIA, however, segmentation is instead regarded as a pre-processing step (Castilla and Hay, 2008), and its results, namely, image objects, are rarely envisaged as end products. The process of endowing the image objects with meaning is a complex one (Castilla and Hay, 2008) and usually takes place in the classification step. From this perspective, automation of the segmentation process is a necessary step toward the automation of image processing in GEOBIA. While some degree of automation in segmentation has been achieved for specific tasks, for example, the extraction of tree-crown objects (Ardila et al., 2012), generic solutions are rare. Esch et al. (2008) developed a segmentation optimisation procedure that was based on spectral similarity between image objects at two scales in a hierarchy. Although MRS was employed to generate the two scales, segmentations were conducted without optimising the data; thus, the results do not directly depend on the segmentation itself but instead depend on the statistics of the arbitrarily-generated parent/children image objects. Drăguţ and Eisank (2012) proposed a concept for automating the optimisation of the SP, which has been successfully applied for automated object-based classification of topography from SRTM data. However, this approach works on a single layer, which hinders applications on multi-spectral data. In brief, a generic solution to automate the parameterisation in MRS is still missing, which is considered to be a disadvantage of GEOBIA (Whiteside et al., 2011) and a priority for further research (Jakubowski et al., 2013).

Building on the work of Drăguţ et al. (2010) and Drăguţ and Eisank (2012), this study introduced a fully automated methodology for the selection of suitable SPs relative to the patterns encoded within the data. Compared to previous approaches, this work considers multiple layers and implements a three-level hierarchy concept. Woodcock and Harward (1992) showed that a single-scale segmentation is an unrealistically simple scene model. On the one hand, some landscape elements are structured in nested hierarchies, for example, a forest composed of forest stands and individual trees (Woodcock and Harward, 1992). This concept is accommodated in eCognition® by building parent/child relationships when choosing the ‘hierarchy’ option in segmentation. On the other hand, visible features in a landscape are often multidimensional (e.g., small buildings coexisting with large agricultural fields), and each feature class is best represented at a certain scale (Martha et al., 2011). This issue is technically tractable by combining image objects of different sizes, which are created with the ‘non-hierarchy option’. In any scenario, multi-scale segmentation is more suitable than single-scale to model image objects in a scene (Woodcock and Harward, 1992).

We demonstrated the performance of the tool in three test cases, on very high spatial resolution (VHR) multispectral imagery, in different applications scenarios. For these applications scenarios, we used expert delineated polygons and quantitative measures (Clinton et al., 2010) to evaluate the results of the segmentations.

## 2 Methods

The methodology comprises the computation of LV on multiple layers (Section 2.2), to allow optimal SPs to be selected automatically (Section 2.3). The workflow was implemented using eCognition Network Language (CNL), within the eCognition® 8.7.2 software, as a customised algorithm that is easy and ready to use (Section 2.4). The final outputs of the tool are assessed using quantitative measures (Section 2.5).

### 2.1 Study areas and data

Various test areas were chosen to assess the behaviour of the tool in diverse situations, ranging from urban settlements to semi-natural landscapes, as described in Table 1. We focused on areas for which we had access to VHR imagery. The first test area (T1) is located in Darfur, Sudan, and covers 2.31 km^2^. It represents a semi-arid Sahel landscape that includes wadis, isolated trees and the Zam Zam internally displaced persons camp. Traditional (dark) huts and bright tents are the main dwelling types in the camp. A QuickBird scene, which was acquired on December 20th, 2004, by Digital Globe, Inc., was used for the T1 area. It includes a panchromatic band at 0.6 m spatial resolution with three visible (RGB) bands and one near-infrared (NIR) band at 2.4 m. The image was pan-sharpened to 0.6 m with the Gram-Schmidt spectral sharpening algorithm (Laben and Brower, 2000).

**Table 1:** Summary of the three test areas and imagery types.

Test	Imagery (all pansharpened)	Spatial resolution (in m)	Number of bands	Coverage (in km^2^)	Landscape characteristics	Location
T1	QuickBird	0.6	4	2.31	Temporary settlements in savanna	Sudan. Zam Zam internally displaced people camp in Darfur.

T2	WorldView-2	0.5	8	2.25	Mixed residential/industrial/agricultural area	Austria. Western part of Salzburg city.

T3	WorldView-2	0.5	8	3.05	Mixed riparian/agricultural area	Austria and Germany. Salzach river zone between Salzburg city and Oberndorf.

The T2 test area covers 2.25 km^2^ in the western part of the city of Salzburg, Austria and includes residential, industrial and agricultural features. The T3 test area represents a semi-natural landscape at the border between Austria and Germany, between the cities of Salzburg and Oberndorf. Extended across 3.05 km^2^ and crossed by the river of Salzach, it includes forests, agricultural fields and water bodies. T2 and T3 are covered by WorldView-2 satellite images that were acquired on September 11th, 2010 (T2) and July 9th, 2011 (T3). The original bands were: panchromatic at 0.5 m spatial resolution and multispectral at 2 m spatial resolution, namely, coastal blue, blue, green, yellow, red, red-edge, NIR 1, and NIR 2. The images were pan-sharpened to 0.5 m with the Hyperspherical Colour Sharpening algorithm implemented within ERDAS IMAGINE.

### 2.2 LV on multiple layers

To take full advantage of the multispectral information, segmentation on multiple layers is desirable. To accomplish this goal, a mean value of LV (*mean*LV) is computed for each image level that was created; the ratio between the sum of the LVs for each layer (LV *1*–LV*n*) and the number of layers (*n*) used in the image segmentation is given in (1):

(1) $$\mathrm{mean}\;\text{LV}=({\text{LV}}_{1}+{\text{LV}}_{2}+\cdots +{\text{LV}}_{n})/n$$

**Fig. 1:**
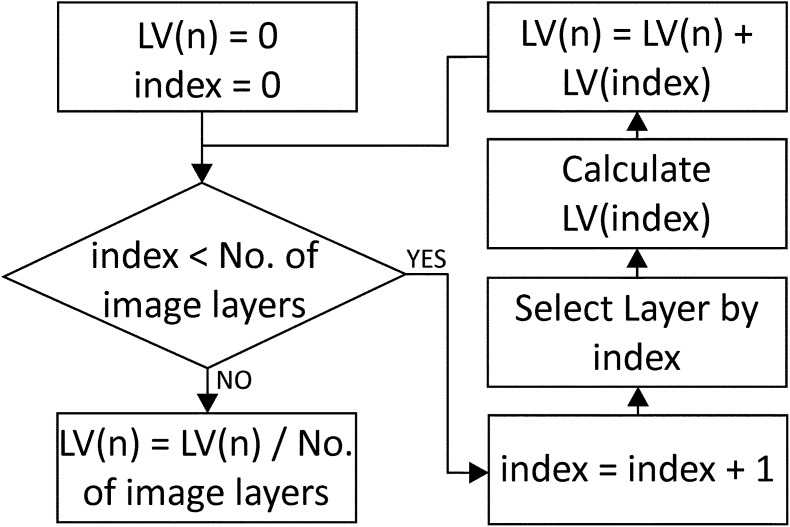
The workflow to compute the mean local variance using a layer index.

The maximum number of layers is not limited because the tool automatically identifies the total number of layers within the scene as well as their names. This computation is implemented through an iterative process, using an index that allows scrolling through all of the layers that are loaded into eCognition®. To derive the mean of LV for the entire scene, each layer is selected; its value of LV is computed, LV(index), and added to the final variable, LV(*n*), which is divided by the total number of image layers present in the project (Fig. 1). The process is executed as long as the index value is smaller than the number of image layers, as recorded during the iteration. It is important to note that all of the layers included in the project are used to segment the scene into image objects. If a user wants to exclude specific layers/multispectral bands from the analysis, the layers can be loaded after the execution of the tool.

### 2.3 Automated selection of SPs

**Fig. 2:**
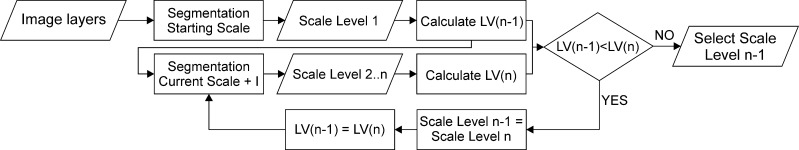
Extracting the scale parameter value that corresponds to the threshold in the LV graph (after Drăguţ and Eisank, 2012). *I* is the lag at which the scale parameter grows.

The automated selection of SPs is basically an automation of the ESP tool, where production of a graph is replaced by an iterative procedure that segments an image at the first threshold that occurs in the LV graph. Drăguţ and Eisank (2012) found that the increment in SP, i.e., the lag at which the SP grows, has powerful control over the scale because it can smooth the LV graph in such a way that prominent peaks at the finest scale turn into first thresholds in coarser-increment graphs. These thresholds can be automatically extracted at the point where the LV value at a given level (LV*n*) is equal to or lower than the value recorded at the previous level (LV*n−1*). The level *n* − 1 is then selected as the optimal scale for segmentation (Drăguţ and Eisank, 2012) (Fig. 2).

Based on this idea, we implemented an automated image segmentation process at three optimal scales, with default scale increments of 1, 10 and 100, following a user-defined option of employing either a hierarchy or a non-hierarchy approach. In a non-hierarchic approach, the MRS algorithm independently creates three levels that start from pixels at each step of the iteration; thus, no parent/child relationship can be established between the image objects that belong to these scale levels. In contrast, the hierarchy option leads to building each level on the image-object that was already created at the previous level, which might be the above level in a top-down approach (i.e., segmenting image objects from broader scales downward) or the finer level in a bottom-up sequence (i.e., merging image objects upward). The user’s decision of choosing one approach or another depends on the purposes of the study.

All of the LV values are recorded in a table that can be exported and processed with the freely available stand-alone software described in Drăguţ et al. (2010). Thus, a user can assess the scales that are detected by the automated version or can simply choose other representative scales according to the specific goal of a project.

### 2.4 Computer implementation

**Fig. 3:**
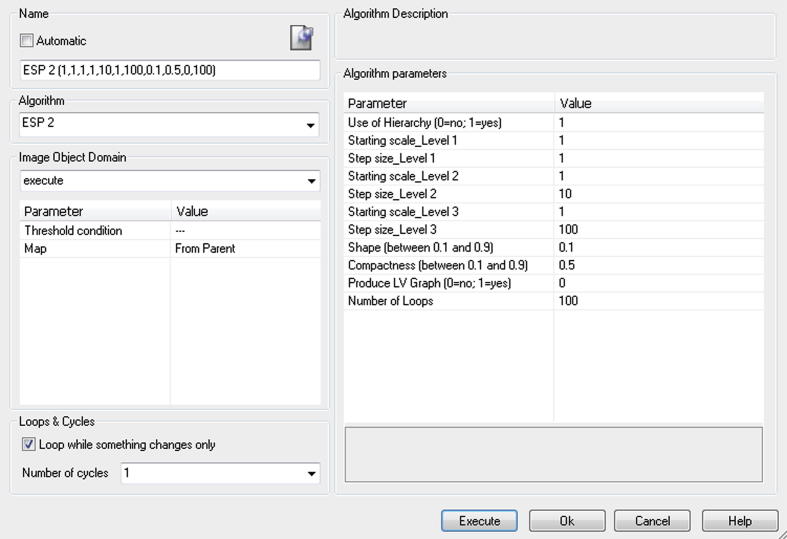
The graphical user interface of the tool, implemented as a process in the eCognition® software. The default variables of the tool (values on the right panel) were used in the tests described below.

The tool was programmed in CNL within the eCognition® software environment. CNL is a modular programming language for handling image objects in a vertical and horizontal hierarchy (see also Tiede et al., 2011). This tool is available as a ready-to-use customised algorithm, which can be seamlessly integrated into CNL to develop rule-sets or used solely for segmentation. Fig. 3 shows the GUI (graphical user interface) of the tool, where additional options for the calculation process can be chosen by a user. Additional options encompass, amongst other capabilities, the selection of a hierarchical or non-hierarchical approach (see Section 2.3), the export of an LV-graph for further analysis and the modification of the MRS weighting for the use of a shape criterion (compactness versus smoothness) in the segmentation process. The step-size and starting scale selection are initially defined (see Section 2.3), but they can be modified as well, according to the specific conditions (e.g., very complex scenes in which some classes of objects might require refinements).

### 2.5 Evaluation of tool performance

To assess the goodness of the segmentations, the outputs were compared to reference polygons that were mostly manually delineated in the images that were analysed (Table 2). A set of metrics proposed by Clinton et al. (2010) were used to quantify the spatial match between reference polygons and individual image objects of the automatically generated segmentation levels. We used a minimum of 50% as a threshold for overlapping objects, which is considered to be appropriate for the problem of matching objects in the assessment of the segmentation goodness (Zhan et al., 2005).

**Table 2:** Summary of segmentation results, reference data, and segmentation accuracy metrics. The references in T1 (L2 and L3) were created in Hagenlocher et al. (2012). SP- scale parameter, AFI- area fit index, US – Under-Segmentation, OS – over-segmentation, D- index combining US and OS, and QR- quality rate.

Test	Segmentation results			Reference data		Segmentation accuracy metrics				
	Level	SP	Number of objects	Number of reference polygons	Source	AFI	OS	US	D	QR
T1	L1	83	5621	50 dwellings	Manual delineation	0.47	0.54	0.12	0.39	0.57
	L2	201	861	50 trees	Supervised classification	0.35	0.49	0.22	0.38	0.56
	L3	301	361	21 wadis	Supervised classification	0.70	0.75	0.17	0.55	0.77

T2	L1	184	3574	224 small buildings	GMES Urban Atlas	0.70	0.79	0.29	0.59	0.80
	L2	371	948	152 buildings	GMES Urban Atlas	0.72	0.78	0.22	0.57	0.79
	L3	701	228	22 fields	Manual delineation	0.28	0.35	0.10	0.26	0.39

T3	L1	224	1536	56 trees, small fields	Manual delineation	0.03	0.09	0.07	0.08	0.15
	L2	441	434	50 tree groups, fields	Manual delineation	0.09	0.17	0.09	0.14	0.24
	L3	701	214	35 water bodies, tree groups, large fields	Manual delineation	0.06	0.10	0.05	0.08	0.14

The following segmentation goodness metrics were computed: Area Fit Index (AFI), Under-Segmentation (US), Over-Segmentation (OS), an index that combines US and OS (D), and the Quality Rate (QR). All of the metrics range from 0 to 1, where 0 indicates perfect spatial match between reference polygons and individual image objects. Details on these metrics (including formulas) can be found in Clinton et al. (2010).

## 3 Results

### 3.1 Segmentation results

For the three tests, the same parameterisation of the tool was applied to the input images (Fig. 3). The three levels were denoted L1, L2, and L3, where L1 represented the finest object scale and L3 the broadest scale. The number of image objects per level decreased accordingly from L1 to L3 (Table 2).

**Fig. 4:**
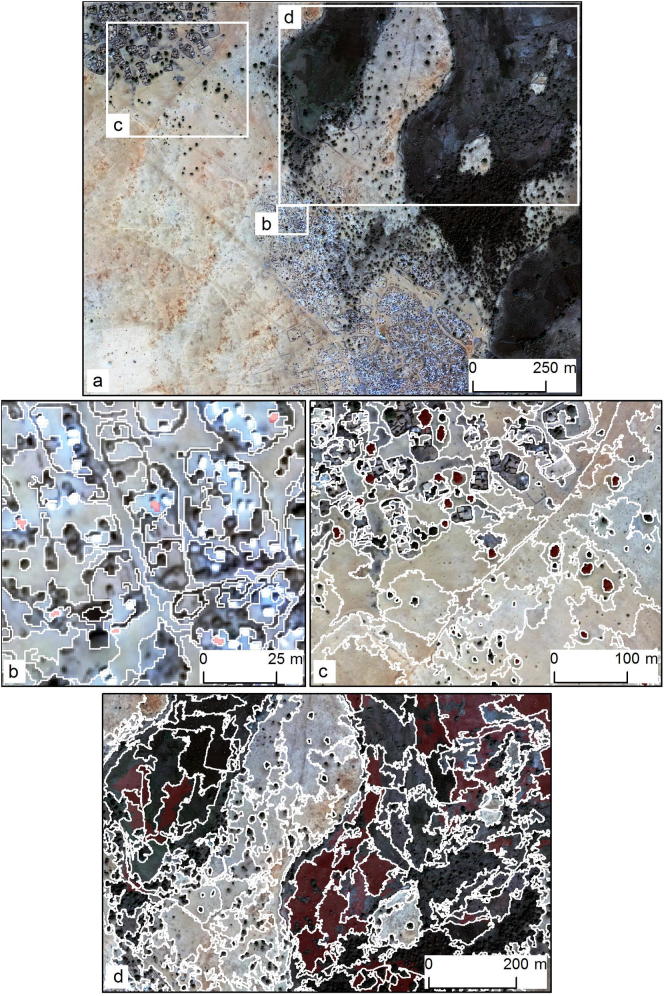
Temporary settlement in savanna (T1): the entire QuickBird image (a) and the subsets used for visualising results (b–d). Segmentation results (white outlines) and reference polygons (solid red) for L1 (b), L2 (c), and L3 (d).

For the temporary settlement area in the African savanna (T1), the tool identified SPs of 83 (L1), 201 (L2) and 301 (L3). The segmentation levels that were generated with these SPs (Fig. 4b–d) partially delineated the representative scales of the image objects as present in the original QuickBird scene (Fig. 4a): individual dwellings (4b; L1), individual trees and clusters of dwellings (4c; L2), as well as wadi structures and vegetation patches (4d; L3). The segmentation results were compared to manually interpreted dwellings (L1) as well as individual trees (L2) and wadis (L3). The last two types of objects originated from a recently performed supervised classification of the same QuickBird image (Hagenlocher et al., 2012).

**Fig. 5:**
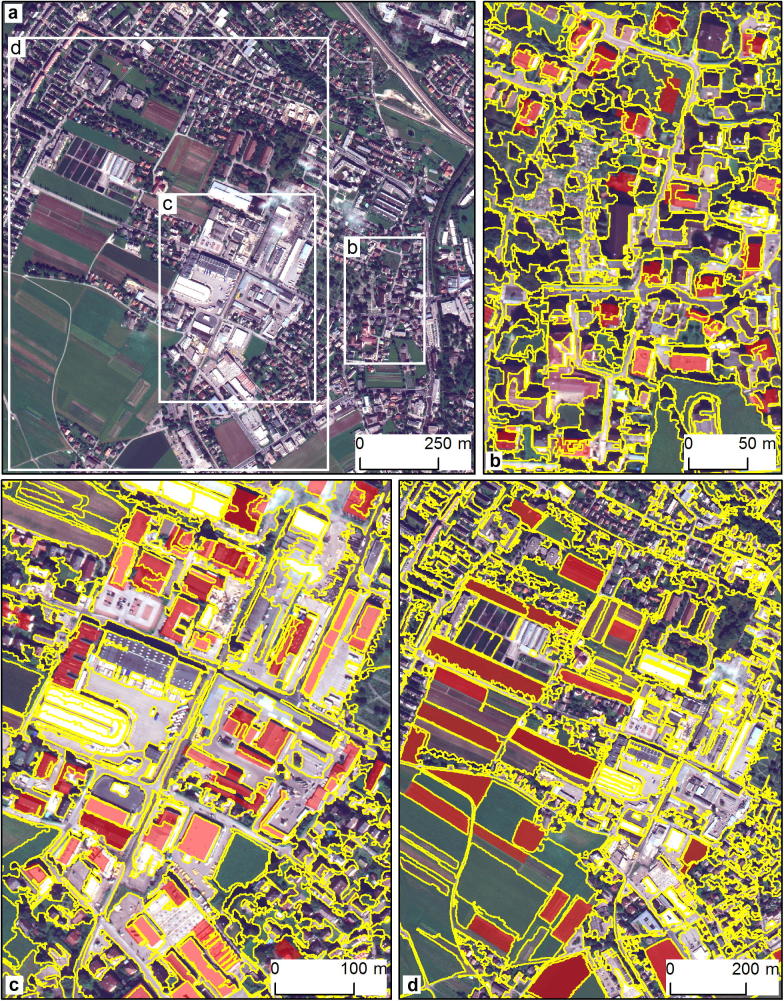
Mixed residential/industrial/agricultural area (T2): the entire WorldView-2 image (a) and the subsets used for visualising results (b–d). Segmentation results (yellow outlines) and reference polygons (solid red) for L1 (b), L2 (c), and L3 (d).

The three identified SPs for the mixed residential/industrial/agricultural area (T2) were 184 for L1, 371 for L2, and 701 for L3. The original WorldView-2 scene and the zoomed versions of the obtained segmentation levels are presented in Fig. 5. Visually, the image objects partially represent similar-sized groups of geo-objects, such as buildings, trees, and open spaces in L1 (Fig. 5b) and L2 (Fig. 5c), as well as agricultural fields and residential areas in L3 (Fig. 5d). The image objects were assessed against reference polygons: the image objects in L1 and L2 were evaluated against polygons that represent buildings, which were included in a freely available GIS land cover dataset for the city of Salzburg (GMES Urban Atlas; http://www.eea.europa.eu/data-and-maps/data/urban-atlas). Manually delineated fields served as references for assessing L3 objects.

**Fig. 6:**
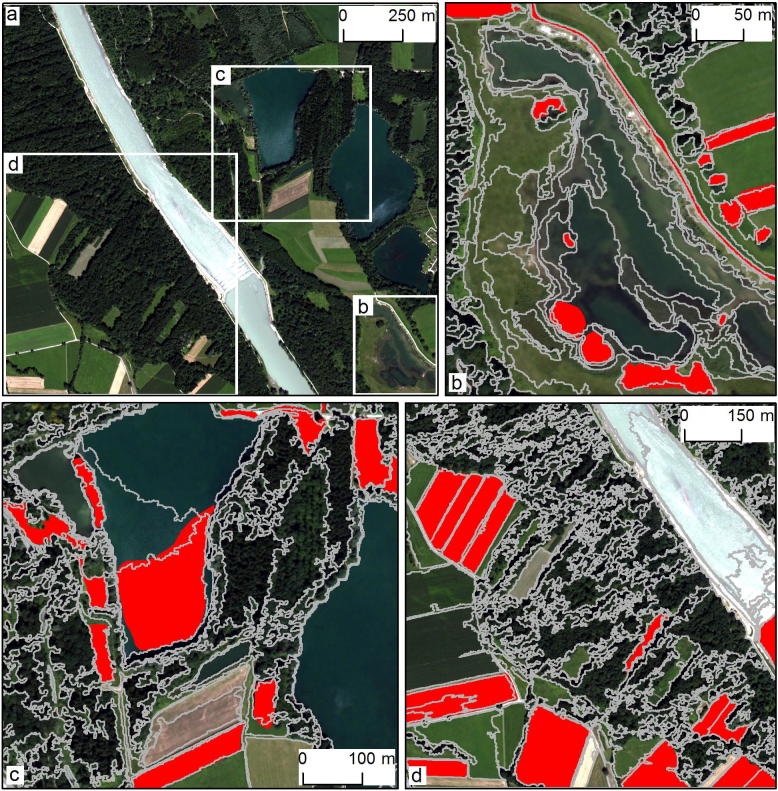
Mixed riparian/agricultural area (T3): the entire WorldView-2 image (a) and the subsets used for visualising results (b–d). Segmentation results (grey outlines) and reference polygons (solid red) for L1 (b), L2 (c), and L3 (d).

The third test was conducted in a mixed riparian/agricultural area. Fig. 6(a–d) depicts selected parts of the three segmentation levels at SPs of 224 (b; L1), 441 (c; L2), and 701 (d; L3), as well as the original WorldView-2 image (a). The image objects in L1 partially delineated single trees, field roads, and small agricultural fields. At L2, groups of trees, small water bodies, and agricultural fields were recognised. The image objects in L3 can be visually associated with large agricultural fields and water bodies as well as with forest patches. Reference polygons were manually interpreted based on the WorldView-2 image and mainly represent the previously mentioned categories of image objects.

### 3.2 Segmentation accuracy

Table 2 provides a summary of the segmentation results, the utilised reference data, and the calculated segmentation accuracy metrics.

The best accuracies were achieved for the mixed riparian/agricultural area in T3. As depicted by Table 2, all of the level-specific metrics were close to 0, which indicates a nearly perfect spatial match between the manually derived reference polygons and their largest intersecting objects. Comparatively, worse accuracies were obtained for the finest and intermediate levels (L1 and L2) in T2 and for the coarsest level in T1 (L3).

In all cases, the value of US was relatively low and ranged from 0.05 (L3 in T3) to 0.29 (L1 in T2). These results mean that most of the image objects did not over-estimate the reference too much, which is desirable. However, the relatively high values of OS, AFI, and QR for T1 and T2 (except for L3) suggest that the reference polygons were usually far larger in size compared to the evaluated image objects.

We further tested the hypothesis that the tool performs at least as well as segmentations with randomly-selected SPs. Thus, we generated 10 random SP values equally stratified (e.g. 1–100, 101–200 and so on) along the interval 1–1000. The segmentation results at these SP values were then evaluated with the same metrics as above (see Section 2.5). The results for L3 in T3 are shown in Table 3. As expected, the number of objects decreased with increasing SP. Except for US, all segmentation accuracy metrics followed the same trend of decreasing with increasing SP until R6 and R7, then increasing as the SP increased. US recorded the minimum value of 0.01 at R2, then stabilized between 0.04 and 0.06. The finest level recorded no US value, as the SP of 30 produced largely over-segmented image objects (which led to an undefined value of D, as calculation of D includes US) (Clinton et al., 2010). As the reference objects for L3 in T3 were quite large, under-segmentation was not an issue for any of the evaluated levels (including the one obtained with the tool). The other segmentation accuracy metrics suggest that SPs along the interval 537–673 would perform relatively equal in matching the reference objects, in spite of the difference in the number of objects (328 vs. 223). However, the tool performed consistently better than any of the randomly-generated segmentations (Table 3).

**Table 3:** Summary of segmentation accuracy metrics for 10 randomly-generated levels (R1 to R10). Stars (∗) denote undefined results. Please refer to Table 2 for abbreviations.

Segmentation results			Segmentation accuracy metrics				
Level	SP	Number of objects	AFI	OS	US	D	QR
T3, L3	701	214	0.06	0.10	0.05	0.08	0.14
R1	30	50833	1.00	1.00	∗	∗	1.00
R2	160	2970	0.87	0.87	0.01	0.62	0.87
R3	210	1787	0.64	0.65	0.04	0.46	0.66
R4	322	782	0.40	0.44	0.06	0.32	0.46
R5	485	388	0.14	0.18	0.04	0.13	0.20
R6	537	328	0.11	0.16	0.05	0.11	0.19
R7	673	223	0.10	0.14	0.05	0.11	0.18
R8	791	167	0.15	0.18	0.04	0.13	0.21
R9	863	145	0.16	0.20	0.04	0.14	0.22
R10	943	118	0.17	0.21	0.04	0.15	0.23

## 4 Discussion

Novelties of this approach are the following: (1) Application of LV on multiple layers; and (2) Automation in the detection of SPs by implementing a three-level hierarchy concept. In previous studies (Drăguţ et al., 2010, Kim et al., 2008), the concept of LV (Woodcock and Strahler, 1987) was implemented on single layers. The adaptation of LVs on multiple layers is less straightforward, however. One way of assessing the homogeneity of the image objects with LV would be to consider the average LV values of all of the layers that are included in the segmentation process. Alternatively, the minimum LV values across all of the layers can be considered to be an indicator of a suitable SP. We tested both of the solutions and obtained results (not shown here) that were slightly better with the latter approach, especially for image objects with poor contrast. While the minimum LV would provide the ‘purest’ definition of the object homogeneity, it increases the time of processing with the number of layers because each layer must be segmented individually, and then, the SPs corresponding to the minimum LV are to be used for the segmentation of all of the layers. We therefore implemented the average LV to define the homogeneity of the image objects on multiple layers.

Automation of the detection of SPs relies on the procedure introduced by Drăguţ and Eisank (2012). In their study, automation was applied to a single layer, which contained the elevation data. Adapting the procedure to perform multi-layer segmentation resulted in the challenge of specifying the number of layers to be considered in the segmentation as well as in the calculation of the average LV value. To address this issue, we implemented an index that allows counting the total number of layers added to eCognition® and considering them all in processing. This solution makes the tool independent of a specific sensor and allows the integration of multiple datasets (e.g., ancillary data). Integration of spectral and ancillary data has been found to be important in an increasing number of recent GEOBIA-related applications. The ancillary data include geo-spatial datasets, such as roads or other vector datasets (Hagenlocher et al., 2012, Verbeeck et al., 2012), airborne laser scanning point clouds (Beger et al., 2011) or their derivatives (Hellesen and Matikainen, 2013, Lu et al., 2011), Digital Elevation Models and/or their derivatives (Hölbling et al., 2012, Lahousse et al., 2011, Martha et al., 2010, Stumpf and Kerle, 2011, Sun et al., 2012), and Digital Surface Models (Aguilar et al., 2012, Shruthi et al., 2011).

The number of levels produced automatically is somewhat arbitrary because appropriate scales partly depend on the objectives of a study (Wiens, 1989). Based on the Hierarchy theory, Hay et al. (2002) suggested a generic three-tiered nested system in the modelling of a landscape structure with remote sensing techniques. This concept was technically implemented by smoothing the LV graph (see Drăguţ and Eisank, (2012) for a detailed explanation of the rationales of this smoothing), with scale lags in three orders of magnitude, specifically, 1, 10 and 100. SPs of higher magnitudes are less likely to occur in typical applications.

Assessing the quality of the image segmentation is difficult because currently no standard evaluation methods exist (Van Den Eeckhaut et al., 2012). The results of segmentation accuracy assessment might look poor, especially for L3 in T1 and levels 1 and 2 in T2. Certainly, the computed accuracy metrics depend on the reference data. In this study, the reference data came from three different sources: manual delineation, supervised classification, and the GMES Urban Atlas. The best accuracies were achieved when the reference polygons were manually mapped; the GMES Urban Atlas references yielded the lowest accuracies. However, visual inspections revealed the fact that errors in the reference data contributed only marginally to the poor accuracy metrics in the above cases. In T1, these results were due mainly to the misfit between the human understanding of ‘wadis’ and the way in which the respective image objects are defined in terms of homogeneity. Looking to Fig. 4d, one can see that the boundaries of the reference data are not visible in the image. This pattern occurs because the study in which the reference data were produced used complex criteria to define wadis, according to the objectives of that study (Hagenlocher et al., 2012). This case is well-suited to illustrate a limitation of the tool: it produces statistically relevant segmentations, which do not necessarily meet a given semantically relevant category of objects. This limitation relates to the semantic gap between image objects and geo-objects (Castilla and Hay, 2008, Eisank et al., 2011), which is still a subject of research (Arvor et al., 2013).

The tool follows the observation that, in hierarchical systems, the variance increases as the scale transitions are approached (O’Neill, 1986). In this approach, a sudden increase in variation, which is generated by a statistically significant occurrence of similar objects (in terms of the size and physical properties), indicates scales at which the between-group differences are especially large (O’Neill, 1986), which suggests a natural scale that is specific to these objects. Therefore, only those image-object categories that follow the pattern encoded within the data and that are well-represented in the study area can be targeted for a ready-to-use segmentation. Semantically complex categories or image objects that are less representative in a given scene should be further processed with a class-modelling approach (Tiede et al., 2010), which couples segmentation and classification in a cyclic process. In such an approach, the initial segmentation results are to some degree not crucial for the derivation of representative image objects, and both under- and over-segmentation can be accommodated.

Levels 1 and 2 in T2 produced low accuracy metrics due to the difference in lighting on the roofs because the reference objects in these two levels were buildings only (Table 2). To assess the generic performance of the tool, rather than its applicability for specific purposes, the images were deliberately not pre-processed. As a result, spectral differences between the sunny and shady sides generated systematic segmentations of roofs into two objects, thus increasing the OS value, which accounts for over-segmentation. A closer look at Fig. 5, b and c reveals that the roof edges were accurately delineated in most cases, however. The most important indicator of segmentation accuracy is US, which accounts for the ‘true’ segmentation error, because the under-segmented areas cannot be resolved further in the classification step (Neubert et al., 2008). In contrast, over-segmented areas can still be merged into desired objects by applying classification rules (e.g., roofs can be re-constructed from their halves, as long as each individual half is accurately segmented). From this perspective, the tool performed very well, with US metrics being always lower than OS and close to 0 (Table 2).

It is worth highlighting that the identified SP values and the obtained numbers of image objects appear to depend on the radiometric resolution, number of bands, and scene complexity. When comparing the results of T1 and T2, in which images of similar spatial extents but different radiometric and spectral resolutions served as input, it turns out that the values of the detected SPs for the same level were higher in T2 (WorldView-2) when compared to T1 (QuickBird). In T2 and T3, the same image type, i.e., WorldView-2, was used. Because the scene in T3 was approximately one-third larger than the scene in T2 (Table 1), the identified SPs in T3 were usually higher (Table 2). However, despite the smaller extent of the T2 scene, far more objects were delineated, especially for L1 and L2. This finding occurred because T2 exhibited a much higher complexity (urban structures) than T3 (a semi-natural landscape). The tool worked in a self-adaptive fashion, accommodating the SP values to these differences.

Time is an important factor when assessing the performance of a tool. The processing times increased with the number of layers, from approximately 20 min (T1; extent of 9.24 mil. pixels) to several hours (T2, T3; extents of 9 and 12.2 mil. pixels, respectively) on a 3.1 GHz quad core station with 8 GB RAM. For an eight-band image, such as WorldView-2, an extent of some 1 mil. pixels appears to be a practical limitation for reasonable processing time. Beyond this limit, the processing time tends to increase exponentially. Of course, tasks on a smaller number of layers can be performed on larger extents. Additionally, the non-hierarchy option slows down the processing because the segmentations are performed directly on the pixels, unlike the hierarchy option, with which higher levels are obtained through merging the already existing sub-objects. Therefore, we recommend choosing the hierarchy option when time is important. In an operational setting, this limitation of the extent can be tackled by applying the automatic tiling and stitching methods that are available with the eCognition Server®. Similarly, the performance of the tool can be improved by masking out irrelevant areas for a given purpose and avoiding *no data* values in the segmentation.

The tool described here has a significant potential of increasing the objectivity and automation in the GEOBIA applications. First, it offers a statistical solution to the decision of selecting the SP values to perform segmentation on multiple layers. Second, the tool can be seamlessly integrated within a CNL-based process tree in eCognition® to automate the workflows. Considering the known limitations of this approach (as discussed above), we do not expect successful automation in any possible case, especially when targeting semantically complex categories of image objects; however, at least first approximations of scales that exist within the data are feasible. Because this tool creates three readily available scale levels, we expect that it will foster especially multi-scale GEOBIA applications, which were found to perform better than single-scale approaches (Kim et al., 2011). Additionally, a handy solution to the spatial scaling of remote sensing imagery might be helpful in gaining further insights into fundamental issues of scale and hierarchy.

## 5 Conclusions

A generic solution for the objective selection of the SPs to perform MRS on multiple layers was missing in GEOBIA. We introduced a fully automated methodology for the selection of SPs to perform MRS at three distinct scales with the eCognition® software. Tests on QuickBird and WorldView-2 imagery provided satisfactory results in three areas, which range from urban settlements to semi-natural landscapes. The tool looks useful as a generic solution for the tessellation of satellite imagery relative to the patterns encoded in the data.
